# Occupational (In)visibility: The emerging role of the Remote Education Tutor as an educational conduit

**DOI:** 10.1007/s13384-022-00576-7

**Published:** 2022-10-07

**Authors:** K. L. Peel, B. McLennan, P. A. Danaher, E. Burnett

**Affiliations:** 1 University of Southern Queensland, Toowoomba, Australia; 2 Australian Geographically Isolated Learner Education, Clermont, Australia

**Keywords:** Credentialling, Distance education, Equity, Geographically isolated students, Governesses, Remote education tutors, Rural and remote education

## Abstract

Remote Education Tutors (RETs) are central to the delivery of distance schooling in Australia and are accountable for the face-to face supervision and educational support of students. They act as the government mandated adult supervisors of Australian primary and secondary school students enrolled in distance education, including geographically isolated learners. This paper draws on statistical data from a national survey (*N* = 575) that was designed to map the perceptions of Australian RETs. These data confirmed that RETs act as a conduit between the distance schooling teacher and student, and that their role requires complex capabilities to be performed within a structured framework. Time restrictions with competing demands present a constant challenge to the RETs’ work satisfaction. Constraining this occupation is the reality that there is no formal qualification available for RETs. Without specific credentialling, it appears that the RETs’ (in)visible role risks being overlooked as a substantive educational occupation.

## Introduction

Schools of distance education in Australia were established historically to provide students in geographically isolated regions access to education (Buckingham, 2017; Roberts & Downes, 2020). Particular locations of rural Australia are geographically remote with widely dispersed populations, leaving distance education or boarding school the only options for students living in these areas (Downes et al., 2020; Newman, 2014). This paper focusses on a research project, specific to rural and remote education, that narrows to an issue of fair and equitable education entitlement for geographically isolated students.

The literature on rural education highlights that related policies and structures play a role in the equitable provision of educational opportunities (Downes & Roberts, 2015; Stokes et al., 1999; Sullivan et al., 2018). A diversity of research has evolved over time to identify the distinctive issues associated with education in Australian rural and remote contexts (Downes & Roberts, 2018). Contemporary research has explored the rural perspectives of the varying aspects of education, as an endeavour to connect *rurality* to the broader educational field (Fuqua & Roberts, 2021; Fuqua et al., 2021). Whilst the distinctiveness of education in rural places is recognised, there is a similarity across the challenges that include several related issues: teacher shortages and staff incentivisation in rural schools (Burke & Buchanan, 2022); attracting and retaining teachers in rural and remote settings (Kelly & Fogarty, 2015; Roberts, 2004); expectations and quality of work life in rural and remote teaching (Sharplin, 2002, 2009); rural placement and teacher education (Kline et al., 2013; White & Reid, 2008); professional development for rural teachers (Broadley, 2010); and leadership in rural schools (Drummond & Halsey, 2013). Within the broader discourse of Australian rural education, it has been noted by some that disadvantage exists in regional and rural schools (Lamb et al., 2014), and that this extends to the students’ experiences of inequality related to educational opportunities (Sullivan et al., 2018).

Regional and rural inequality in distance schooling arises from the significant cost to geographically isolated families, who through necessity are required to educate their children via a distance education programme (Chesters & Cuervo, 2022; ICPA, 2020). Given the low density populations of remote locations it often means that it is not practical for school authorities to establish schools in these areas (Downes et al., 2020); geographically isolated students engaging in distance education are required to be at a physical distance from the nearest school location. This is authorised to be more than 56 kms from the family home; in reality, it is often hundreds of kilometres (Services Australia, 2022). Hence, the delivery of this mode of instruction relies on both synchronous and asynchronous delivery (Roberts & Downes, 2020). Given the complexity of curriculum delivery and the accountability of children attending school (Newman, 2014), there is a requirement that children enrolled in Australian schools of distance education have adult supervision during their school day.

The delivery of this distance education curriculum cannot be achieved without the commitment of the Remote Education Tutor (RET), who is accountable for the face-to-face adult supervision and educational support of distance education learners (Downes et al., 2020; ICPA, 2015; Newman, 2014). This almost exclusively female workforce of home based educators includes two distinct groups: mothers or *parent tutors*, who supervise their own children’s education, known as *immediate family tutors;* and *governesses* who are *externally employed* in a paid supervisory position (McLennan et al., 2022). The RET role is crucial for distance education students, yet there is little research to understand this occupation (Downes, 2013; Newman, 2014).

More specifically, RETs act as facilitators and conduits, who mobilise the partnerships that are integral to the teaching and learning processes. The events of the recent COVID-19 pandemic have shone a light on the challenges of learning remotely from home. Yet, what is not well known is that, for many children, education delivered in the home is the norm rather than a response to exceptional circumstances (Roberts & Downes, 2020). The significance of the research project presented in this paper resides in its acknowledgement of the tangible existence of the RET occupation, and the complexity of the role that they are required to play as the home based educator of distance schooling students. Further, the study was designed to recognise the personal and professional impacts on lifestyles of this essential work and, in turn, to raise the profile of this position as an occupation identified and valued by the broader population. Moreover, this often overlooked, underestimated and undervalued occupation (Downes, 2013) is crucial to ensuring the equity of children’s access to consistent and quality educational support. Furthermore, it can be argued that the recognition of this workforce would also contribute to the quality of education that leads to the sustainability of rural and remote communities and populations (Commonwealth of Australia, 2020; Downes & Roberts, 2015).

This paper presents the first of multiple phases of intended research that contributes to an understanding of who it is that represents the RET workforce in Australia, and how they self-report their personal and professional perceptions about this work. The multi-phase design adopted a mixed method approach whereby Phase 1 was developed as a three part survey that included items about demographics and two measures:

- Part A. Australian RET workforce demographics;
- Part B. RETs’ Personal and Professional Perceptions; and
- Part C. RETs’ Basic Needs Satisfaction in the Work Domain, a published, standardised test (adapted from Chen et al., 2015; Schultz et al., 2015).

Initial findings of demographics from the 2021 nation-wide survey (*N* = 575) encapsulated four key elements about the limited career pathway for the RET, the associated economic disadvantage from living in a remote setting, the expectation that the mother fulfils the supervisory requirement as the RET in the schoolroom, and the equitable provision of affordable and accessible educational expertise (McLennan et al., 2022). The balance of the findings from these survey data and the implications are discussed in this paper, where the participants self-report about their personal and professional perceptions of their practices, and their work satisfaction, given the position of being an RET.

## Background

Beyond the urban centres of cities and towns across Australia, where the country is densely populated, there exist families in geographical locations where the children require remote access to education via distance schooling. Evidence about the provision of distance education has raised significant community debate in relation to the level of satisfaction with its accessibility and affordability (Human Rights & Equal Opportunities Commission, 2000). The distance education schools and their qualified teachers are responsible for organising and administering the curriculum for students (Harris et al., 2022), who are often located hundreds of kilometres from where the teaching is generated.

The responsibility for setting up a dedicated space as a formal schoolroom and the expectation that a supervisor will oversee a child’s learning reside with the families. The literature has highlighted the importance of a partnership between the distance education teachers and the families to ensure seamless teaching for quality learning outcomes (Harris et al., 2022; Lee & Wilks, 2007). However, research indicates that the opportunity for quality distance education is unsustainable because of the limited supply of employees to work in RET positions (Douglas, 2019). Further, there is evidence of inequity in financing education, such as insufficient government subsidies to assist parents to fund the supervision of their children’s learning (ICPA, 2020), and a lack of credentialling opportunities for the role that RETs are required to play (Newman, 2014).

An *Independent Review into Regional, Rural and Remote Education* (Halsey, 2018) was commissioned by the Commonwealth Department of Education and Training, where Halsey concluded, “The key challenge for regional, rural and remote education is ensuring, regardless of location or circumstances, that every young person has access to high quality schooling and opportunities” (p. 1). It is accepted that rural and remote communities confront unique challenges to ensuring equitable education (Dockett & Perry, 2021; ICPA, 2018; Roberts et al., 2018; Sullivan et al., 2013).

Limited literature is available on the demographics and the work of the RET (Newman, 2014; Pini & Mills, 2015), whose (in)visible work encompasses the identities of a *Governess*, *Home Tutor*, *Parent* or *Family Tutor* or *Distance Education Tutor*. Previously, a study was conducted across distance education school stakeholders to research the role of distance education tutors in supporting literacy and numeracy development, and the use of technology in early education (Dole et al., 2005; Lee & Wilks, 2007). However, this research was located in a different era, prior to the online learning platform developments in current distance schooling (Harris et al., 2022). Very few studies have focussed on the position of the home based educator who works in remote education. This is despite a 1999 recommendation by the Queensland School Curriculum Council (1999) to conduct explicit research into this position, and a report that identified the considerable expense that supervising children for a school day placed on the families accessing distance education (Stokes et al., 1999). A responsive study by Green (2006) gained insights of everyday life in the schoolrooms of families enrolled in distance education. Almost a decade ago, Downes (2013) investigated the supervisory role of parents in primary school distance education. Newman (2014) undertook an inquiry to seek better understanding of the educational work of governesses in outback Australia. Concerningly, researchers (Green et al., 2013) reported on the tendency to treat home tutors from a deficit perspective rather than valuing their knowledge and experience, and that mothers acting in the role were under pressure to ensure the completion of all the prescribed daily tasks on schedule.

For varied reasons, many families whose children are enrolled in distance schooling do not employ a governess in the position of the RET (McLennan et al., 2022). Parents, and specifically mothers, by default, take on the RET supervisory responsibility; often feeling obliged to fulfil this complex and sometimes incompatible position (Downes & Roberts, 2015; Newman, 2014; Tynan & O’Neill, 2007). The maternal role of mothers, in combination with the mandated supervision of their children’s education, has the potential to manifest as relationship tension (Downes & Roberts, 2015; ICPA, 2015). However, the societal assumption that mothers are available to provide educational supervision is shifting in concert with broader, contemporary social changes, and has been described as no longer valid (Alston & Kent, 2008; ICPA, 2020). Importantly, recent research, as part of this study, has been conducted to address this gap in the literature about the demographics of RETs in Australia (McLennan et al., 2022).

In changing times and varying circumstances, some families elect to outsource the RET position. However, the limited supply of RETs and the lack of funding to employ someone to fulfil this position often mean that families are under significant pressure to provide adequate education for their children, if this is the path that they choose. Moreover, the complexity of the role and the requirements of flexible, knowledgeable, and relational facilitators to carry out effectively this crucial connective position (Downes, 2013; Lee & Wilks, 2007) raise further concerns about the accessibility of quality distance education. Of note is the fact that there is currently no prerequisite qualification requirement specifically tailored to RETs in Australia (Downes, 2013; ICPA, 2018).

Much of the existing information about primary and secondary distance schooling in Australia has been sourced by the Isolated Children’s Parents’ Association of Australia (ICPA), which exists as a voluntary body dedicated to ensuring equitable access to education for geographically isolated children (ICPA, 2018, 2020). A recent ICPA (2020) submission to the Inquiry into *Education in Remote and Complex Environments* indicated that access to affordable and appropriate educational services is a major factor in determining whether families will remain in geographically isolated locations. Accordingly, an increasing number of families are vacating rural areas and relocating to larger cities and towns to gain equitable, affordable education for their children (Commonwealth of Australia, 2020; Corbett, 2007). This is clear evidence that families are forced into difficult decisions and situations, and that the sustainability of Australian rural and remote communities is consequently threatened. The key to achieving high-quality educational opportunities for all children living in geographically isolated areas is the availability of proficient RETs, who act as the educational conduit between the distance education teacher and the student (Downes, 2013).

It follows, then, that the value of the RETs’ position inherent in a distance education setting must be acknowledged through formal recognition of their skills and credentials, and through suitable remuneration and subsidisation (ICPA, 2018). The formal recognition of the RETs’ prerequisite skills would provide status as an occupational incentive for those considering this career pathway. This was articulated in the 2018 government submission (ICPA, 2018): “A formal recognition of the role of the distance education tutor and the skills acquired would provide incentive for those considering employment in this role” (p. 18). To help to influence the general issues pertaining to the RETs’ role within distance schooling that have been outlined, it was imperative to investigate the very people who undertake this work and their perceptions of their experiences (Newman, 2014).

## Conceptual framework

The study presented in this paper is located in the social cognitive paradigm, which is an accepted theoretical perspective in educational research. That is, the reciprocal interactions that are experienced by the RETs in their workplace suggest that the perceptions of their work fulfilment are a consequence of personal, behavioural and environmental influences (Bandura, 1986). Social cognitive theory recognises the interplay among the thought processes and feelings, the observable behaviours and the environmental events in explaining why RETs’ perceptions of their work are situationally specific and context dependent.

**Fig. 1:**
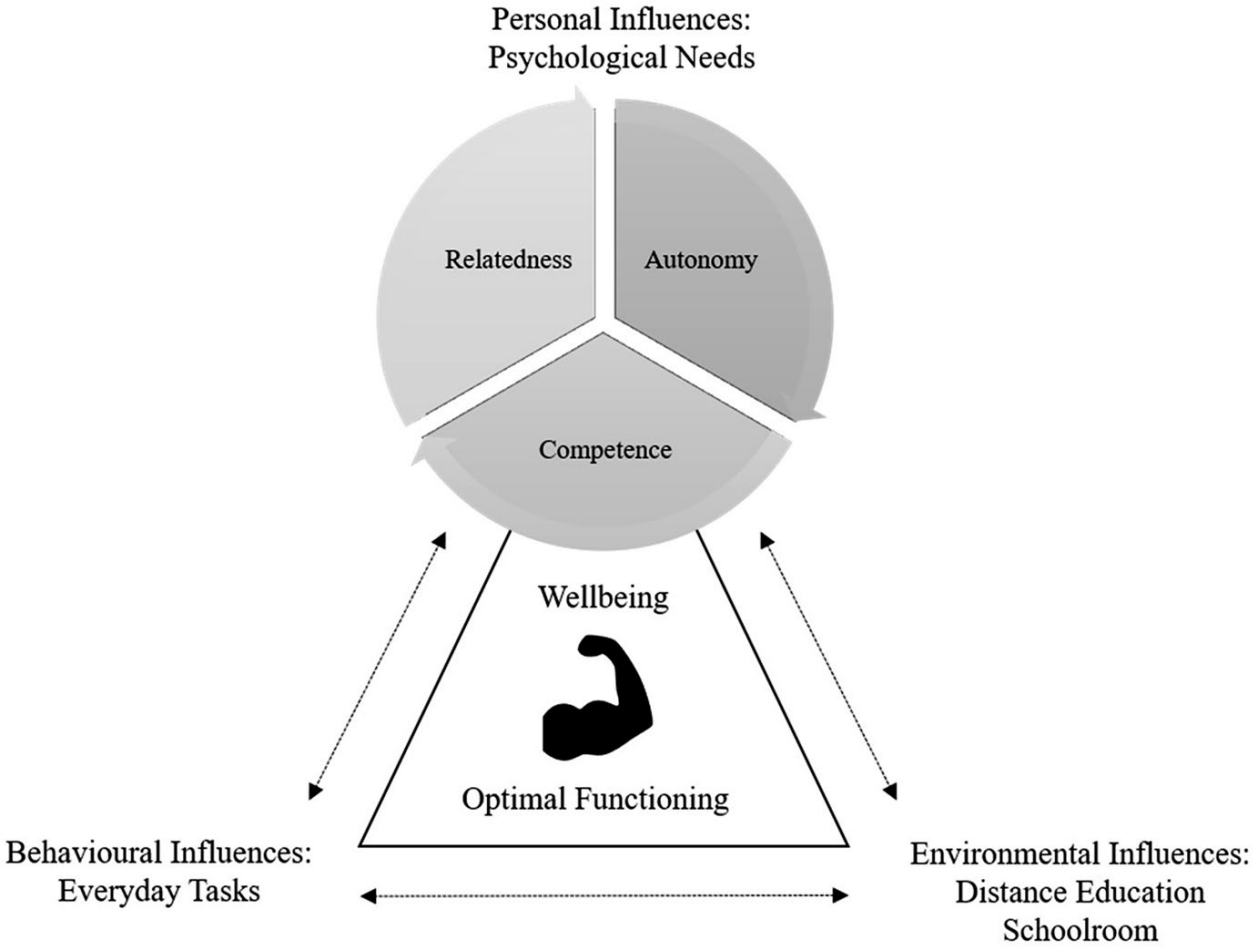
The conceptual framework adapted from Bandura’s (1986) triadic reciprocal causation model, including the psychological needs (Ryan & Deci, 2002)

The personal influences in the adaptation of Bandura’s (1986) triadic reciprocal causation model are represented in this project as the psychological needs, which integrates self-determination theory (Ryan & Deci, 2002) into the conceptual framework. The continuum of motivation from the theory of self-determination (Deci et al., 2017) represents the sources of influence that extend from extrinsic to intrinsic motivation. This motivational continuum is useful to illustrate the satisfaction (or frustration) with regard to the psychological needs. Thus, personal influences include the satisfaction or frustration of autonomy, competence and relatedness as the psychological needs that determine their performance and how this perception of performance informs their subsequent actions. Environmental influences, derived from the distance education context and the social interactions that occur within it, impact on the RETs’ opportunities to engage purposefully in their work. Behavioural influences, pertaining to the RETs’ requirement to perform their work, impact on the ways that they respond through their strategic actions and decisions. In Fig. 1, the arrows diagrammatically illustrate the interactions connecting these three key influences.

### Personal influences

Self-determination theory posits that social contexts promote increased internalisation and intrinsic motivation when these contexts satisfy the psychological needs of autonomy*,* competence and relatedness (Ryan & Deci, 2002). To the extent that the perceived satisfaction of needs is perpetually met, individuals will grow and function effectively and experience wellbeing. Conversely, the extent to which these needs are thwarted will make it more likely for individuals to experience ill-being and non-optimal functioning.

Additionally, self-determination theory purports that all humans contain an intrinsic need to be self-determining, feel competent and be connected to others, allowing them to be fully functioning within their immediate environment (Ryan & Deci, 2002). Autonomy, competence and relatedness, as personal influences, serve as integral ingredients of the conceptual framework of this project. For RETs, autonomy is promoted through having choices and volition, and opportunities to self-initiate behaviours. Their competence is enhanced when situated in a connected learning setting, affording optimal challenge and informational performance feedback, affirming and stimulating their proficiency or mastery. Relatedness for RETs emanates from building interpersonal relationships within a culture of care, empathy, and a strong sense of belonging. Beyond the RETs’ personal perceptions of relatedness, they need to be acutely aware of the interconnectedness of the multiple stakeholders who exist in the workplace environment that work towards the common goal of educating the child.

### Environmental influences

The RET is committed to work with the children in the schoolroom, following a structured routine and timetable, in the online and offline spaces (Capricornia School of Distance Education, 2020). There is an expectation from the schools of distance education that the schoolrooms will replicate in many ways what a child would experience in a mainstream school classroom (Downes, 2013). The learning spaces are therefore required to be designed to provide work areas that are adorned with instructional posters, other artefacts of student learning, and that are generally free from unnecessary distractions (Green, 2006; Stokes et al., 1999). The schoolroom has provision for Internet access and a laptop for the children’s engagement in online learning and offline task completion.

The environment in which the RET works also includes a virtual space, where regular contact with the children’s teachers is maintained in a timely manner. The cyclical relationship between the distance education teachers who provide the guidelines, and the RET, who shares the learning outcomes, is essential for the success of the children’s learning (Downes, 2013). Similarly, when the RET is externally employed by the family of the child, a further relationship is to be nurtured. The distance education context, and the social interactions within it, also create the platform for a unique relationship between the RET and the child (Tynan & O’Neill, 2007).

### Behavioural influences

The work of RETs has been classified into four role categories according to the types of everyday tasks carried out in the schoolroom (Downes, 2013; ICPA, 2015). The four categories include organise, communicate, teach and manage (McLennan et al., 2022). These categories align with the practices that were identified by Downes (2013), who researched the role of parental supervision in geographically isolated locations. The demands of the tasks inherent in the categories represent behavioural influences that impact on the RETs’ perceptions of their workplace. The ways in which they respond to the demands of the tasks influence their decision-making and strategic actions in future experiences. Table 1 presents the RETs’ role categories and the corresponding everyday tasks.

**Table 1:** The role categories and everyday tasks of RETs (McLennan et al., 2022)

Role categories	Everyday tasks
Organise	Setting up and maintaining a well-organised and resourced classroom
	Ensuring that students link to the Internet for their formal instruction
	Receiving and returning school resources
	Receiving and returning completed work from and to the distance education school
	Planning and timetabling
	Marking schoolwork before it is returned
Communicate	Interacting with parents, teachers and schools in relation to learning
Teach	Supervising the support work to complement the online lessons
	Providing specific numeracy instructional support
	Providing explicit reading and writing instruction
	Delivering and providing support for other curriculum learning areas
	Planning interventions for learning difficulties
Manage	Managing disciplinary issues
	Keeping students engaged in the curriculum during their online lessons

Through the conceptual framework designed for this project, it is evident that social cognitive theory provides a fitting lens through which to investigate the (in)visible occupation of the RET in distance schooling. This camouflaged occupation, ubiquitous by its multiple labels, is not invisible to those intimately involved; however, there is undoubtedly a distinct lack of rigorous academic analysis to raise general awareness of this undervalued occupation (Downes, 2013; Green, 2006). The research elaborated below was designed to investigate how the participants self-reported their personal and professional perceptions of their practices, and their work satisfaction, given their role of being an RET.

## Research design

This multiple phase research project used a sequential, investigatory, mixed-methods approach that was endorsed by Creswell and Clark (2013) as practical, in that the researchers had a full complement of methods available to resolve research problems. The first of the multiple phases, as reported in this paper, incorporated a research survey for the purpose of investigating a group of people, by asking a series of questions and tabulating their responses (Leedy & Ormrod, 2013). This approach enabled a large sample size to be investigated efficiently to form generalisations about the group and their experiences within a distinctive educational context.

### Context and participants

The participants who were recruited for a 2021 Australia-wide survey consisted of both previously and currently engaged RETs. Of the total participants in this study (*N* = 575), the currently practising RETs (*n* = 339) were divided into two groups: the immediate family RETs (*n* = 207); and the externally employed RETs (*n* = 132). The RETs were recruited across Australia through industry partnerships such as distance education jurisdictions and ICPA state and federal organisations. The participants were from all Australian States and Territories, except Victoria and the Australian Capital Territory. Queensland (69.88%) had the largest representation across Australia, and the Capricornia School of Distance Education contributed the most responses (27.20%) in that state. Of the participants in the survey, the majority of the currently practising RETs in the position identified as working in geographically isolated locations.

The participants in this survey identified themselves as being in the position of *Parent Tutor, Governess/Govie, Home Tutor, Distance Education Tutor, Family Tutor, Nanny* or *Home Teacher.* The most prevalent self-descriptions of the position were *Parent Tutor* (37.91%) and *Governess* (37.74%). Of the total participants, 59.48% were currently practising as RETs, of whom 87.72% located themselves in the geographically isolated educational category. The participants were predominantly working in the primary school sector (81.68%). Across all participants, the RETs’ ages were reported as being between 31 and 50 years, with the currently externally employed RETs aged predominantly between 20 and 30 years. The overwhelming gender of all the participants was female at 99.13%, and all the immediate family RETs were the mother of the child/ren whom they supervised.

### Data collection

On reading the participation information form and agreeing to engage in the research survey, the participants were instructed to click the survey link that confirmed their consent. The survey was estimated to take 20 minutes to complete, and participation in this study was voluntary and anonymous. All the ethical considerations from the university (Ethics approval: H20REA214) were observed.

The survey was constructed using *LimeSurvey*, an online statistical survey tool. Beyond questions related to the demographics of the workforce (*N* = 575), it consisted of two measures. The first was an investigatory measure (*N* = 534), whose items emanated from organic sources, such as the literature about the complex role of RETs (Downes, 2013), informal conversations with RETs, professional discussions with leaders in the distance education sector and various online media. The purpose of this piloted measure was to gain insight into RETs’ personal and professional perceptions of their practices, given the distinctiveness of their position in distance education. The second measure (*N* = 498) was an established instrument: Basic Psychological Need Satisfaction and Frustration Scale (BPNSFS)—Work Domain (adapted from Chen et al., 2015; Schultz et al., 2015). This identified RETs’ self-perceptions of autonomy, competence and relatedness regarding their satisfaction and frustration of these needs at work.

#### Personal and professional perceptions scale

The Personal and Professional Perceptions Scale (PPPS) provided a questionnaire as an organic measure developed to assist in the analysis of the insights of the unique position of RETs. The scale consists of 18 items, given as nine descriptors each with two items: comfortability, accountability, social wellness, contentedness, resilience, belonging, productivity, volition and versatility. The items are phrased in general terms of how participants perceive the impact of their work as RETs and are indicated by a 5-point Likert scale. The respondents self-reported how true it was for them on a scale from 1 (strongly disagree) to 5 (strongly agree) that their role of the RET impacted on their personal and professional perceptions of their practices. Examples of the items are found in Table 2.

**Table 2:** Descriptors and example items in the personal and professional perceptions measure

Descriptors	Example items
Temporal Comfortability	My lifestyle and living arrangements allow time for myself
Accountability	My responsibility for facilitating a quality teaching and learning experience is empowering
Social Wellness	One advantage of being an RET is that it includes unique and varied experiences
Contentedness	My RET role is rewarding and fulfilling
Resilience	My work requires me to adapt, face challenges and overcome adversity
Belonging	I build positive relationships in the workplace that are easy to maintain
Productivity	Supportive and accessible resources in the schoolroom allow me to engage in effective teaching and learning
Volition	I often have the opportunity to direct my work to meet the expected educational outcomes for the children
Versatility	Being adaptable and flexible adds to the positive experience of being an RET

#### Basic psychological need satisfaction and frustration scale: work domain

The Basic Psychological Need Satisfaction and Frustration Scale (BPNSFS)—Work Domain (Chen et al., 2015; Schultz et al., 2015) is a measure used to assess needs satisfaction and frustration at work. The scale consists of 24 items, clustered around six subscales: autonomy satisfaction (four items); autonomy frustration (four items); competence satisfaction (four items); competency frustration (four items); relatedness satisfaction (four items) and relatedness frustration (four items). Items are phrased in general terms of how participants perceive need-fulfilment in “their life” and are indicated by a 7-point Likert scale.

As the present study concerned RETs’ need-fulfilment within the context of distance education, the items were adapted from the original scale (Chen et al., 2015; Schultz et al., 2015) to reflect this context. For example, “At work, I feel a sense of choice and freedom in the things I undertake” has been adapted in the new research with minor vocabulary alterations made to ensure cultural and contextual fit. For instance, “At work” was translated into “As an RET”.

Respondents indicated on a Likert scale from 1 (*strongly disagree*) to 7 (*strongly agree*) the extent to which the psychological needs of autonomy, competence and relatedness were generally satisfied in their RET experience. *Autonomy satisfaction* is having a strong sense of volition and psychological freedom, whereas *autonomy frustration* concerns feelings of obligation and pressure to behave in certain ways (Chen et al., 2015; Schultz et al., 2015). Examples of items were as follows: “I feel that my decisions as an RET reflect what I really want” (autonomy satisfaction); and “Most of the things I do as an RET I feel like I have to [do]” (autonomy frustration). *Competence satisfaction* is an increased sense of effectiveness and mastery, as opposed to *competence frustration*, which is an eroded confidence and motivation that could lead to passiveness, avoidance type behaviours and perceived incapableness (Chen et al., 2015; Schultz et al., 2015). Examples of items were as follows: “As an RET, I feel capable at what I do” (competence satisfaction); and “I feel disappointed with my performance in my work” (competence frustration). *Relatedness satisfaction* is a heightened sense of closeness and connection with significant others, whilst *relatedness frustration* is the feelings associated with being alienated or isolated or even lacking a sense of belonging among others in a context (Chen et al., 2015; Schultz et al., 2015). Examples of items were as follows: “I feel that the people I care about at work also care about me” (relatedness satisfaction); and “I feel that people who are important to me at work are cold and distant towards me” (relatedness frustration).

### Data analysis

The analysis of the data from the online survey was situated within the conceptual framework as the measures represented the reciprocal interactions of the personal, behavioural, and environmental influences (Bandura, 1986) that were experienced by the RETs in their workplace. Each of the two measures was analysed separately using a similar process, given that the PPPS is a piloted measure, and the BPNSFS is an already established motivational measure.

**Fig. 2:**
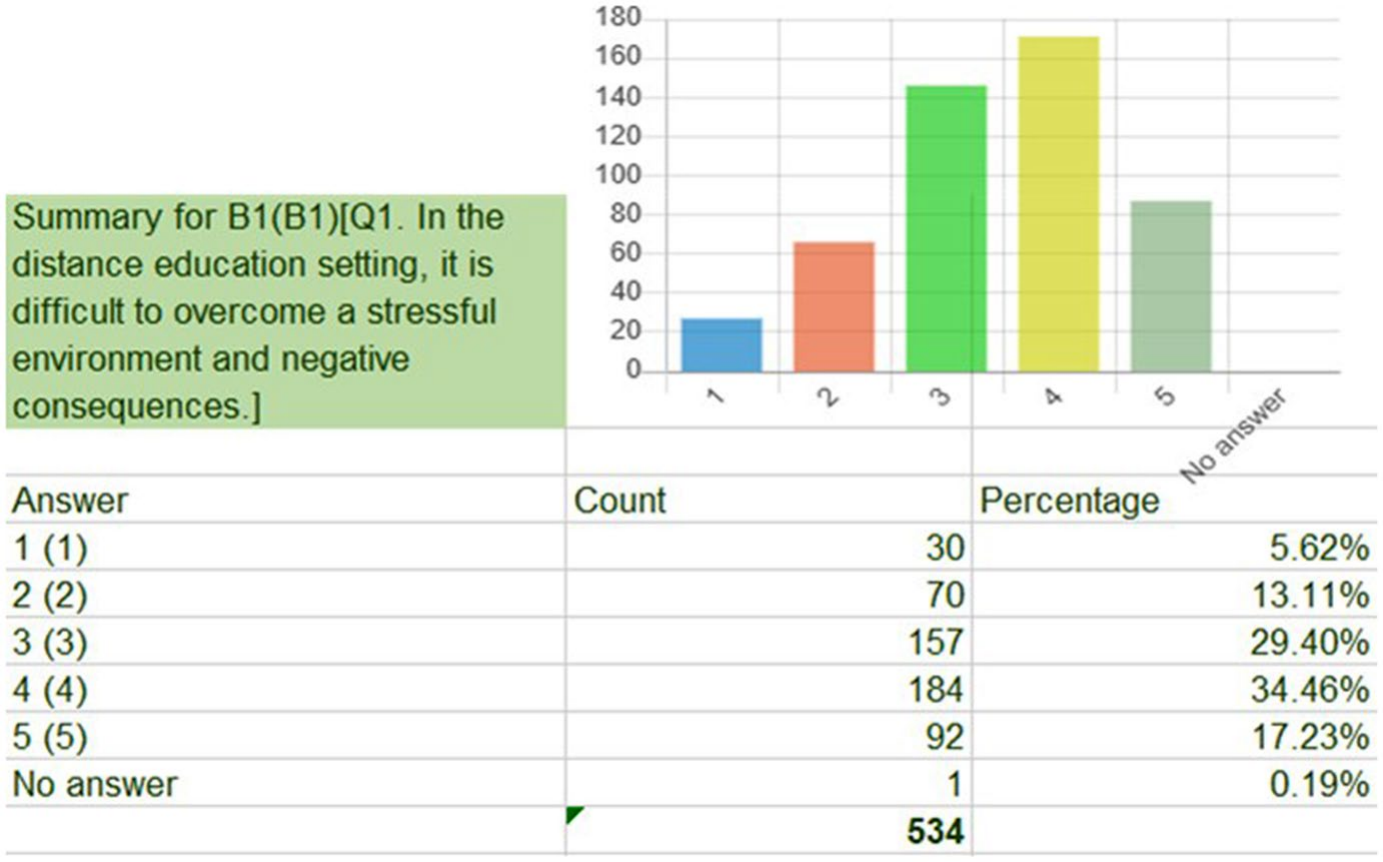
An example of a survey item’s data represented by a percentage table and a related vertical bar graph

The data set from the PPPS was described according to the nine underlying descriptors that acted as the selected lenses through two distinctive items. The data from each item were represented as a percentage table and a related vertical bar graph. Figure 2 shows an example of how each item’s data were examined.

Patterns emerged through the Likert scale of 1 (strongly disagree) to 5 (strongly agree) that represented the participants’ responses to the items. These data were transitioned into descriptive statements. For example, in responding to the item (B1) about RETs overcoming a stressful environment and negative consequences in the distance education setting:

- 29.4% (*N* = 534) declined to state an opinion.
- Over half (51.69%) (*N* = 534) agreed that it was difficult to overcome workplace challenges.

**Fig. 3:**
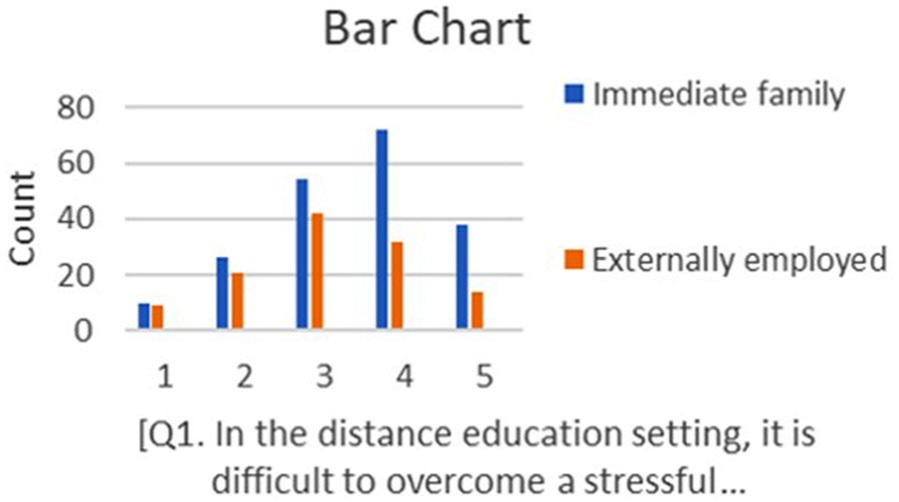
An example of cross-tabulated data for a survey item represented by a vertical bar graph

Furthermore, the data set was then separated according to the status of the participants in relation to whether they were currently practising RETs or not. From this newly established data set (*N* = 318), the participants were identified as being in one of two groups: those working with their own children as immediate family, as opposed to those employed to work with unrelated children as externally employed tutors. Using the *IBM SPSS V27* statistical software, a cross-tabulation analysis of the responses from these two groups of participants was employed. This comparative analysis was represented by graphs and explained through descriptive statements. Figure 3 provides an example of a cross-tabulation graph conducted between the responses of the currently practising immediate family members and the currently externally employed tutors to the item (B1) that was described as the following:

- 55% of currently practising immediate family respondents (*n* = 200), as opposed to 39% of currently externally employed respondents (*n* = 118), stated that it was difficult to overcome workplace challenges.

The descriptive statements, as the key findings, were then each contextualised to generate a proposition (P) that was specifically related to the item. This added value to the discourse around the RETs’ perceptions of how their work impacts on their personal and professional lives. For example, in response to the item (B1), the following was proposed:

P: The currently practising immediate family RETs have reported a significantly higher propensity for experiencing difficulty in overcoming workplace challenges than the externally employed RETs.

Each of the propositions was collated according to its relevant descriptor, with the intention to add significant organic knowledge and understanding to the original descriptor definition.

The data set from the BPNSFS measure was described according to the six factors that each contained four items to make up their respective subscales. Like the PPPS analysis, the data set from each of the BPNSFS items was represented as a percentage table and a related vertical bar graph. Patterns emerged, this time through the Likert scale of 1 (strongly disagree) to 7 (strongly agree), that represented the participants’ responses to the items. These data were again transitioned into descriptive statements.

Replicating the steps in the previous analysis, the data set was separated according to the status of the participants in relation to whether they were currently practising RETs or not. In this specific data set (*N* = 303), the currently participating participants were separated into the same two groups as in the PPPS analysis (immediate family; externally employed), with the comparative analyses’ findings represented by graphs and explained through descriptive statements. These descriptive statements assisted in generating propositions that were specifically related to the basic psychological needs’ items and illuminated the RETs’ perceptions of their work satisfaction and frustration in their workplaces.

## Findings

As conceptualised in Fig. 1, the RETs’ perceptions of their work were situationally specific and context dependent, and it was the interplay across the environmental events that influenced how they behaved when carrying out their everyday tasks in the schoolroom. In addition, the personal influences on the RETs’ thought processes and feelings, such as their satisfaction and frustration in having their psychological needs met, impacted on their perceptions of their personal and professional lives. In order to make visible the crucial nature of the RETs’ role in distance schooling, it was essential to understand this reciprocal relationship for RETs to be fully functioning within their unique work environment. The findings from the survey data are presented here according to each of the measures.

### Personal and professional perceptions

From a total of 534 RETs who participated in this measure for the national survey, 318 reported as currently practising, consisting of two groups. In the first group, 200 participants (of whom all were mothers) identified as immediate family of the students. In the second group, 118 participants identified as external employees.

Nine descriptors were deductively investigated through the Personal and Professional Perceptions Scale (PPPS) for the RETs. In Table 3, a summary of the significant findings is presented, in which each of the descriptors has been defined in the first instance and complemented with data that are represented as descriptive statements. Of note, this table includes data from all the current and past practising RET participants (*N* = 534), and the separated cohorts of the currently practising immediate family RETs (*n* = 200) and currently practising externally employed RETs (*n* = 118).

**Table 3:** Summary of descriptive statements representing the RETs’ personal and professional perceptions of their practices

Descriptors of the PPPS that are defined in the research	Descriptive statements		
	All RET participants: current and past practising (*n* = 534)	Currently practising immediate family RET only (*n* = 200)	Currently practising externally employed RET only (*n* = 118)
*Temporal comfortability* Meeting the time demands of the work	57.3% worked excessive hours	67% worked over reasonable hours	39.82% denied the work required excessive hours
*Accountability* Being responsible for work to meet expectations	71.53% empowered to take responsibility for teaching	74% agreed or strongly agreed meeting the educational demands was overwhelming	
*Social wellness* Feeling fulfilment through social interactions at work	86.33% either agreed or strongly agreed the role presented a varied experience 71.73% were neutral or disputed that isolation was detrimental to wellbeing		76.27% indicated the role provides varied experiences
*Contentedness* Feeling a sense of satisfaction with work	77.9% agreed or strongly agreed the role of RET was satisfying	70.50% reported a level of satisfaction	87.28% reported a level of satisfaction
*Resilience* Being able to adapt to meet the demands of work	86.89% recognised requirement to be adaptable and resilient in the face of challenges	55% stated it was difficult to overcome workplace challenges	39% opposed the idea it was difficult to overcome workplace challenges
*Belonging* Feeling a sense of place in the work environment	66.85% built positive relationships in the workplace that were sustainable		
*Productivity* Being able to engage to meet the work demands	77.15% agreed having the resources in the schoolroom optimised teaching 75.84% reported limited access to technology made teaching challenging		
**Volition** Being able to self-direct the work strategically	37.27% reported a neutral response to being able to direct the work	39.50% perceived opportunities to direct the work	63.55% perceived opportunities to direct the work
*Versatility* Being able to apply an array of skills in the complex work environment	87.08% agreed the role required agility and flexibility to adapt to situations 87.26% agreed the role expectations were complex and required diverse capabilities		

Propositions emanated from the data to challenge notions about the reality of the (in)visibility of the RETs in the distance schooling model. The variance that existed between the two groups’ perceptions of their *temporal comfortability* was inferred to result from the fact that paid employment has set hours that structure the workday, and as such leave appropriate hours to the individual for recreation. Notwithstanding, the immediate family RET had a personal investment, as the mother of the child, and an implied obligation that could lead to going beyond the bounds of a structured workday (Tynan & O’Neill, 2007). Consequently, when the immediate family RET was not separated from the family life, the role consumed time without clear limits (Downes, 2013). By contrast, the externally employed RETs had clear paid time parameters that defined when they entered the school domain and left at the conclusion of the school day.

Despite the overwhelming nature of the work, the RETs reported mostly a sense of empowerment when in the role of teaching. However, for a mother, as the RET, teaching their children increases the level of *accountability* for their education and therefore heightens the burden which places “great weight on the role” (Downes, 2013, p. 32). Green (2006) and Tynan and O’Neill (2007) highlighted that the pressure of teaching one’s own children increased the level of responsibility for their education progress.

With regard to *social wellness*, the participants generally reported a feeling of fulfilment from their varied experiences through their interactions at work. Notwithstanding, professional isolation can impact, positively or negatively, the RETs’ confidence and satisfaction. Interestingly, the perception of professional isolation has been likened to the experience of first-year teachers who have reported feeling unprepared for, and adaptive to, the demands of their work (Kelly & Fogarty, 2015). Externally employed RETs indicated volition in choosing their work and planned their tenure for a defined boundary of time (usually one to two years) that optimised the experiences in the role (Duffy et al., 2015). For externally employed RETs, the availability of employment exceeds the supply of employees (ICPA, 2020). With this excessive demand of employers, it has been inferred that the employees had an advantage of an expanded choice of a desired workplace location.

The RET participants across the board generally self-reported *contentedness* as they felt a sense of satisfaction with their work. Significantly, the externally employed RETs have made informed decisions to take on the role (Newman, 2014), and as such their perceptions were mostly that the role was rewarding and fulfilling. With regard to RETs in the immediate family group, the counterbalance of their obligation to accept the responsibilities, versus the inherent personal reward and fulfilment that they experienced, could explain the higher frequency of neutral responses to their work satisfaction (Downes, 2013).

RETs generally indicated being *resilient* to adapt to meet the demands of their work. It was noteworthy that the immediate family RETs reported a significantly higher propensity for experiencing difficulty in overcoming workplace challenges than their externally employed counterparts. As supported in research by Downes (2013), it was inferred that this occurred because the immediate family RET had multiple roles to be performed across the varying contexts.

The majority of all RET participants felt a sense of *belonging* in their work environment through the positive relationships they formed. Additionally, the perceptions of all of the participants were varied in relation to whether they felt isolated from social support according to their experiences in specific contexts. The variation of responses by externally employed RETs can be explained in the research of Newman (2014), who described the nuances of living among a family, and the challenges associated with separating the education work and the affective labour. It stands to reason that the immediate family RETs were neutral about the perception of belonging, as they were working for themselves in their domestic environment.

Across all of the RET participants, there was a predominant sense that the accessibility to the learning tools ensured work *productivity*. Specifically, the availability of contemporary information and communication technology was perceived to be an enabler for the continuity of teaching and learning in remote education settings (Ames et al., 2021). Technological advancement of communication in distance education makes use of laptops or computers for video conferencing, with reliable access to the Internet service for many RETs working with families in remote communities, remaining one of the greatest barriers (Roberts & Downes, 2020).

Relating to *volition*, all RET participants indicated that there were inconsistencies between having opportunities for directing one’s own work and being directed by others, across the diversity of their role. This was supported by a high rate of neutral responses of the participants to feeling empowered to direct their work. The currently externally employed RETs’ perceptions of empowerment to direct their own work was higher, and it is purported that this difference in perception can be explained through them having the confidence to vary from the scripted lessons distributed by the distance education teachers (Roberts & Downes, 2020), which was not reflected as strongly by the immediate family RET participants.

The RETs self-reported *versatility* to indicate their application of an array of skills in their complex work role (Downes, 2013). Across the groups of RET participants, the attribute of being adaptable in work situations aided in heightening work satisfaction. Surprisingly, for a concealed occupation, or one that is not well understood (Newman, 2014), the participants overwhelmingly perceived that the role of the RET requires a range of capabilities that indicates the position should be recognised as fair work, and also necessitates a level of credentialling (McLennan et al., 2022). Downes (2013) proports that RETs are “an often-overlooked cohort of educators who are essential to education in rural Australia” (p. 32).

### Basic psychological need satisfaction and frustration

The personal satisfaction of the RETs’ basic psychological needs (Ryan & Deci, 2002) is the cornerstone of motivation that interacts reciprocally with the behavioural and environmental influences on their work (Bandura, 1986). From a total of 498 RETs who participated in this measure for the national survey, 303 reported as currently practising, consisting of two groups. In the first group, 191 participants (of whom all were mothers of the children) identified as immediate family. In the second group, 112 participants identified as external employees.

Six factors were deductively investigated through the Basic Psychological Need Satisfaction and Frustration (BPNSFS) adapted for RETs. In the summary of the findings below, each of the factors has been defined in the first instance and complemented with data that are represented as descriptive statements. Propositions emerged from the data to elucidate assumptions about the reality of the (in)visibility of the RETs in the distance schooling model.

In Table 4, a summary of the significant findings is presented, in which each of the factors has been defined in the first instance, and complemented with data that are represented as descriptive statements. Of note, this table includes data from all the current and past practising RET participants (*N* = 498), and the separated groups of the currently practising immediate family RETs (*n* = 191) and currently practising externally employed RETs (*n* = 112).

**Table 4:** Summary of descriptive statements representing the RETs’ work satisfaction and frustration

Factors of the BPNSFS	Descriptive Statements		
	All RET participants: current and past practising (n = 498)	Currently practising immediate family RET only (n = 191)	Currently practising externally employed RET only (n = 112)
Autonomy satisfaction	38.55% remained neutral about being empowered in their role	38.21% agreed they had volition in their role	61.60% agreed that they did have volition
Autonomy frustration	63.05% agreed or were neutral about their daily responsibilities being an established sequence of events 55.42% self-reported feeling compelled to comply with meeting the demands of their role	58.11% indicated their work activities felt like chains of obligations	23.21% indicated that their work activities felt like chains of obligations
Competence satisfaction	72.89% reported feeling capable to meet the demands of their role 77.31% reported being able to complete challenging tasks 64.46% agreed that they felt competent to meet their work goals 74.10% reported feeling confident they could meet the demands of their role		
Competence frustration		51.30% were contented with their performance 54.97% expressed some doubt about their confidence to meet the work demands 40.31% indicated insecurity about their abilities	83.03% were contented with their performance 33.33% expressed some doubt about their confidence to meet the work demands 22.52% indicated insecurity about their abilities
Relatedness satisfaction	72.69% reported positively their perceptions about reciprocal care for those in the workplace 61.44% reported feeling comfortable with the people around them at work 12.04% reported they disagreed that they had positive connections with the people in their workplace		
Relatedness frustration	57.30% reported feeling they did belong in the workplace 71.49% did not have the impression the people with whom they worked disliked them 11.25% reported they agreed to feeling they had superficial relationships at work		

RETs perceived *autonomy satisfaction* when they committed to an activity whereby the source of control of their behaviour came from within. This is consistent with SDT’s continuum of motivation whereby the greater the internal source of control of motivation, the more likely the increased level of satisfaction (Deci, 2017). A significant proportion of all the RETs, who responded to the survey, were neutral about feeling empowered in their jobs, and this was likely to have been because the decisions made in the workplace were context specific, and accordingly, varied depending on the nature of the situation (Mazzetti et al., 2021). Given this, it is assumed that the respondents were reluctant to position themselves either way with regard to empowered decision-making.

It was noteworthy that currently practising externally employed RETs reported greater volition in their decisions about work activities and were more comfortable with the choices that they made in their role than their immediate family counterparts. This in part speaks to the positively correlated relationship that exists between autonomy and satisfaction (Deci et al., 1996). It is proposed that the externally employed RETs had purposefully selected a pathway for the work, whilst the immediate family RET had an obligation to fulfil the role (Downes, 2013). That is, one group had chosen to take on the role, and the other group felt compelled to take it on. Additionally, the immediate family RETs were likely to feel that it was important to follow the distance education system as it was prescribed. This motivation is represented along the continuum (Deci et al., 2017), whereby the RETs’ behaviour is driven by their identification that an activity is significant.

RETs felt *autonomy frustration,* when implementing an activity, where the source of control of their behaviour was externally imposed or coercively pressured (Chen et al., 2015; Longo et al., 2016; Schultz et al., 2015). The RETs had indicated that they generally accepted that their role had guidelines within which to work that provided the expectations and role descriptions. This again is represented on the continuum of motivation (Deci et al., 2017) where an individual identifies with the importance of an activity inherent to their work. Given this, it is understood that consistent professional development was required to ensure the RETs could continue to deliver on their expectations and role descriptions. It is suggested that this emphasised the need for a career pathway (McLennan et al., 2022) that could be developed through credentialling (Stokes et al., 1999). Importantly, some immediate family RETs felt compelled to accept the role, whilst others felt it was their responsibility to do so. Irrespective, the immediate family RETs were more likely to be susceptible to carrying the burden of educating their child. Further, the immediate family RETs would also be likely to indicate that their work was part of their daily routine, as a chore. By contrast, the externally employed RETs did not see the work as an obligation, as they were not a family member connected to the child/ren. Therefore, they were not as susceptible to the inherent pressure and subsequent introjected source of motivation as that of a parent (Deci et al., 2017).

RETs perceived *competence satisfaction* when they felt capable of achieving positive outcomes and knowing that they had the capabilities to do so again (Ryan & Deci, 2002). Largely, RETs self-reported feeling confident that they could meet the varied demands of their role, and that they were well equipped to complete challenging tasks (Green, 2006). In part, this can be explained through their indication that they were clear about the expectations of their role and felt that these expectations were attainable. It follows, then, that RETs felt enabled to set goals independently with outcomes that were measurable to identify their competency. Yet there are no formal industry credentials that frame standards or qualifications to acknowledge this proficiency (McLennan et al., 2022). Therefore, it is assumed that the clear structure and expectations self-reported about their work role were imposed by the external industry agencies. It is noteworthy that it was the currently externally employed RETs who represented significantly higher perceptions of capability. Furthermore, it is also purported that this was due to the role being somewhat an occupation as paid work, versus the blurred immediate family/parental role (Newman, 2014).

RETs perceived *competence frustration* when they felt that they had failed or experienced doubts about their capabilities to complete an activity successfully (Chen et al., 2015; Longo et al., 2016; Schultz et al., 2015). Of the current RETs, the externally employed group indicated that they were satisfied with their work to a greater extent than the immediate family. In addition, the externally employed RETs self-reported that they experienced less uncertainty about whether they could fulfil their role in comparison to immediate family RETs, who expressed heightened doubt. This was likely because of the burden of responsibility that immediate family RETs carried when supervising their own child’s education (Downes, 2013). There was a perceived responsibility of the immediate family RETs that came from the inherent pressure that they felt about the obligation to provide their own children with a quality education in the distance schooling setting. The immediate family RETs would most likely consider themselves as a parent first, and a tutor second, whereas the externally employed RETs had a distinctive role in a paid position as the tutor. It is proposed that the larger neutrality in feelings of doubt about their performance shown by immediate family, as opposed to externally employed RETs with regard to competence, centred on what can be described as the blurred role of the mother as an RET (Downes & Roberts, 2015; Newman, 2014). The challenge for the parent to reconcile the tension between being the caregiver and the educator cannot be underestimated.

RETs experienced *relatedness satisfaction* when they felt that they belonged, had purpose, and felt a genuine connection with others in distinctive interpersonal contexts (Ryan & Deci, 2002). The majority of all the RETs who responded to the survey perceived that they worked in situations that could be best described as harmonious with regard to relationships. Most respondents expressed that they felt a caring connection with the people in their work context, with only a small proportion of all RETs indicating a disconnect. It is worth noting that these connections included students and distance education teachers for the immediate family RETs (Downes, 2013; Green, 2006) and extended to the property owners for the externally employed RETs (Newman, 2014). It should be recognised that the externally employed RETs self-reported a similarly positive connection to the immediate family RETs, given that they were not related as a family member.

RETs experienced *relatedness frustration* when they felt excluded and isolated, which likely led to loneliness in distinctive interpersonal contexts (Chen et al., 2015; Longo et al., 2016; Schultz et al., 2015). Notably, most RETs who responded to the survey generally reported a strong sense of personal connection to the significant people in their workplace. Immediate family and externally employed RETs conclusively reported that they did not feel alienated in their workplace, as they had made an informed decision to work in the distance education context. There was a strong feeling that the workplace was harmonious without evidence of perceived fractured relationships. Very few of the respondents surveyed agreed to feeling that they had superficial relationships in the workplace.

## Discussion

This research aimed to identify who represents the RET workforce in Australian distance schooling and to understand how they perceive their experiences, in this role, as impacting on them personally and on their professional practices. This discussion draws together the survey findings to represent how the reciprocal interactions of personal, behavioural and environmental influences (Bandura, 1986) explain the RETs’ experiences in their workplace. The triadic framework provides the theoretical perspective to report the RETs’ perceptions of their work fulfilment that are a consequence of their activity in everyday tasks within the context of the schoolroom. The personal influences are identified through the psychological needs that integrate self-determination theory (Ryan & Deci, 2002) into the conceptual framework. The personal satisfaction of the RETs’ basic psychological needs is the cornerstone of motivation that interacts reciprocally with the behavioural and environmental influences on their work.

The RETs are committed to the work of organising, communicating, teaching and managing, as their everyday tasks to support student learning in the schoolroom. Their perceptions of their work and how they respond to the demands of the tasks are impacted by the behavioural influences that are directing their decision-making and strategic actions. The findings indicated that the RETs accepted that their work required a complexity of capabilities, including a high level of adaptability when performing significant teaching tasks (Downes & Roberts, 2015). This justifies the call for strengthening the external recognition of the RET occupation that involves working in an educational role (Newman, 2014). Suitable access to the required learning tools enabled the RETs to achieve outcomes with students, and this correlated with their reported high level of confidence. This competence satisfaction expressed by the RETs indicated their sense of effectiveness and mastery. As an outcome of personal mastery experiences (Bandura, 1997), the RETs conveyed generally an optimistic perception about their capability of achieving positive outcomes in their complex role. It was noteworthy that the immediate family RETs implied, through an indication of neutrality, that they likely had some reservations about their capability to meet the expected outcomes. This perhaps could indicate a degree of competence frustration that has the potential to erode the RETs’ confidence and motivation, and lead to passive compliance (Ryan & Deci, 2002). Previous studies in the field also reported mothers’ feelings of uncertainty and guilt about meeting the distance education school’s expectations (Downes & Roberts, 2015). Alternatively, the externally employed RETs expressed less uncertainty about their capability to meet the expected outcomes, intimating an expectation of success (Bandura, 1997), even though they were indisputably fulfilling an educator’s role (Downes, 2013).

Furthermore, the RETs reported that they had opportunities to be autonomous, where they perceived they have the capacity in their work to direct their behaviour (Ryan & Deci, 2002). However, the work of the home based educator, even though similar to that of a teacher (Downes et al., 2020), required compliance with a structure determined by the distance education school. The RETs’ personal experience of volition and the perception of control over their behavioural decisions indicated a level of autonomy satisfaction, whereas autonomy frustration represented their feelings of obligation and pressure to behave in certain ways. For the externally employed RETs, the sources of autonomy to fulfil their role were internalised, but the sources of motivation of the work could be external in nature. The experience of feeling autonomous varied for the immediate family RETs on a sliding scale of internalisation according to the sources of their motivation (Deci et al., 2017). The RETs’ levels of motivation along the continuum ranged from the role being a burden, feeling an obligation, existing as an identified daily chore, or to being volitionally integrated into daily life. The continuum of motivation represents their autonomy satisfaction/frustration that is influenced through their work in the rural and remote contexts within the distance education framework. Both immediate family and externally employed RETs indicated predominantly that they worked within a framework of clear expectations and with distinct role descriptions. Moreover, collectively the RETs perceived that their autonomy varied according to contextual specifics. Generally, the RETs indicated that they were empowered in their work, yet earlier research reported an undervaluing and underestimating of the RETs’ knowledge and experience (Downes, 2013; Downes & Roberts, 2015; Green, 2006) with the skills of the RETs described as deficient (Green et al., 2013). However, the immediate family RETs with the dual roles of being the mother and the RET reported feeling overwhelmed at times by the demands of the responsibilities, more so than the externally employed RET. Comparably, Roberts and Downes (2020) purported that some parents were concerned that their children could be disadvantaged if they were unable to support them appropriately in the schoolroom.

The RETs’ workplace is shaped by the environmental influences that include the social interactions existing within the schoolroom and the distance education context. The RETs identified a high level of fulfilment in their work context and indicated that this largely depended on the positive social interactions that they encountered. Previous research attributed the partnership between the distance education staff and the RET to the success in the programme (Stokes et al., 1999). The degree of social support experienced determined the level of inclusiveness that the RETs perceived. Relatedness satisfaction for RETs is the heightened sense of closeness and connection they have with significant others. Both the immediate family and externally employed RETs reported a strong feeling that the workplace was harmonious and caring, with few reporting the feeling associated with relatedness frustration through not being part of something or even lacking a sense of belonging with others in the context. The RETs indicated a strong sense of belonging and personal connection to the significant people in their workplace. They also largely agreed that the time demands required for their role were challenging. Previous research indicated that RETs prioritised their role in the schoolroom over their other responsibilities (Roberts & Downs, 2015). The imposition of time constraints can be frustrating for the RETs, as this inhibits their inherent desire to be fully functioning in their role.

## Limitations as recommendations

Although the survey was administered widely, the data represented only a sample of Australian RETs, with an uneven distribution of participants owing mostly to the comparative vastness of Australia’s states and territories. This point in time survey contained selected measures only, and future surveys could extend on this with a greater diversity of relevant instruments. The Personal and Professional Perceptions Scale (PPPS) is an organic measure that was piloted for the first time in the survey instrument relevant to this study. As such, this fledgling instrument provides an opportunity for further statistical analysis to ascertain its validity via factor analysis and its utility across other work roles.

The already established Basic Psychological Need Satisfaction and Frustration Scale (BPNSFS) affords opportunities for future statistical analysis between the two identified groups of immediate family RETs as unpaid workers and externally employed RETs as paid workers. It is recognised that the survey results provided trends, generalities and anomalies. However, it is accepted that deeper understanding of the work of the RETs would benefit through in-depth case studies. Therefore, it is recommended that the data from this survey inform future interview protocols to gain a clearer picture of the RETs’ role that explains what they do, how they do it and why.

## Conclusion

This research confirmed that RETs act as a conduit between the distance schooling teacher and the student, and that their role requires complex capabilities to be performed within a structured framework, despite that role’s general invisibility. Therefore, recalling the study’s conceptual framework outlined in this paper, the empowered RET accepts the high-level responsibility of the role that is performed with fluctuating degrees of confidence. The sources of motivation of the work can be internal or external in nature, as the reasons for acting in the role are circumstantial, ranging from being burdensome through to volitional. The personal connection and care in the RETs’ workplace are undeniable, and contribute to a strong sense of belonging. With the provision of the necessary learning tools, RETs are enabled to meet the obligations of their work with confidence. However, time constraints with competing demands present a constant challenge to the RETs’ work satisfaction.

Whilst the majority of these RETs do not have teaching credentials, nor are studying to be a teacher, they are indisputably fulfilling an educator’s role. Despite this, there is no formal qualification available for RETs to pursue that is designed to support them specifically in their role. Without prerequisite qualifications, it appears that the largely invisible role of the RET risks being overlooked as a substantive educational occupation, yet it is a mandated position by government authorities for distance schooling. Moreover, the findings of this phase of the research project clearly advocate for the long overdue recognition of the crucial role of the RET in Australian distance education.
